# Inhibition of SARS Pseudovirus Cell Entry by Lactoferrin Binding to Heparan Sulfate Proteoglycans

**DOI:** 10.1371/journal.pone.0023710

**Published:** 2011-08-22

**Authors:** Jianshe Lang, Ning Yang, Jiejie Deng, Kangtai Liu, Peng Yang, Guigen Zhang, Chengyu Jiang

**Affiliations:** State Key Laboratory of Medical Molecular Biology, Institute of Basic Medical Sciences, Chinese Academy of Medical Sciences, Peking Union Medical College, Tsinghua University, Beijing, People's Republic of China; University of Minnesota, United States of America

## Abstract

It has been reported that lactoferrin (LF) participates in the host immune response against Severe Acute Respiratory Syndrome Coronavirus (SARS-CoV) invasion by enhancing NK cell activity and stimulating neutrophil aggregation and adhesion. We further investigated the role of LF in the entry of SARS pseudovirus into HEK293E/ACE2-Myc cells. Our results reveal that LF inhibits SARS pseudovirus infection in a dose-dependent manner. Further analysis suggested that LF was able to block the binding of spike protein to host cells at 4°C, indicating that LF exerted its inhibitory function at the viral attachment stage. However, LF did not disrupt the interaction of spike protein with angiotensin-converting enzyme 2 (ACE2), the functional receptor of SARS-CoV. Previous studies have shown that LF colocalizes with the widely distributed cell-surface heparan sulfate proteoglycans (HSPGs). Our experiments have also confirmed this conclusion. Treatment of the cells with heparinase or exogenous heparin prevented binding of spike protein to host cells and inhibited SARS pseudovirus infection, demonstrating that HSPGs provide the binding sites for SARS-CoV invasion at the early attachment phase. Taken together, our results suggest that, in addition to ACE2, HSPGs are essential cell-surface molecules involved in SARS-CoV cell entry. LF may play a protective role in host defense against SARS-CoV infection through binding to HSPGs and blocking the preliminary interaction between SARS-CoV and host cells. Our findings may provide further understanding of SARS-CoV pathogenesis and aid in treatment of this deadly disease.

## Introduction

Severe acute respiratory syndrome (SARS) is an infectious disease that emerged in Guangdong Province, China in November 2002 [1]. This deadly disease rapidly spread to many countries around the world, with a mortality rate of approximately 10%. SARS presents as an atypical pneumonia that often leads to acute respiratory distress syndrome and respiratory failure, the main cause of death [2]. This unusual infectious disease has become a major threat to public health and social stability since its outbreak. To date, there is no effective therapeutic strategy or preventive vaccine available for the treatment of SARS.

In April 2003, a novel coronavirus, SARS coronavirus (SARS-CoV), was identified as the etiological agent of SARS [3]. SARS-CoV is an enveloped, positive-strand RNA virus. Its large RNA genome is approximately 30000 nucleotides in length and encodes a nonstructural replicase complex and structural proteins, including spike (S), envelope (E), membrane (M) and nucleocapsid (N) proteins [4]. Spike protein is the envelope protein responsible for invasion of host cells. Further three-dimensional structure analyses suggest that spike protein is composed of two subunits: S1, which mediates SARS-CoV binding to receptors on host cells, and S2, which triggers virus and host cell membrane fusion [5]. Angiotensin-converting enzyme 2 (ACE2), a metallopeptidase, has been identified as one of the functional receptors of SARS-CoV and is responsible for binding to spike protein and mediating SARS-CoV entry into host cells [6]. Crystallographic studies have shown that a segment containing amino acids 318–510 of S1 is the critical receptor-binding domain for the ACE2 receptor [6]. ACE2 is highly expressed on human lung alveolar epithelial cells, enterocytes of the small intestine and the brush border of the proximal tubular cells of the kidney. These locations of ACE2 expression are consistent with the tissue tropism and pathogenesis of SARS-CoV infection [7]. Other coreceptors or cellular molecules may be required to facilitate SARS-CoV invasion.

During SARS-CoV infection, a host immune response against the virus is triggered. The innate immune response plays an essential role in the inhibition of viral infection. It has been reported that many genes involved in the innate immune response, such as those encoding lactoferrin (LF), S100A9 and Lipocalin 2, participate in SARS-CoV clearance [8]. Among these up-regulated genes, LF expression was elevated by approximately 150 fold in SARS patients compared with healthy controls. That study demonstrated that LF exerted its function in the process of SARS-CoV infection by enhancing NK cell activity and stimulating neutrophil aggregation and adhesion [8]. However, the bioactivity of lactoferrin is not completely understood.

LF is a multifunctional protein present in external secretions, including saliva, tears, milk, nasal and bronchial secretions, gastrointestinal fluids and urine mucosal secretions, and is an important constituent of the neutrophilic granules of leukocytes [9]. LF possesses strong antiviral activity against a broad spectrum of RNA and DNA viruses, such as Sindbis virus [10], cytomegalovirus [11], [12], herpes simplex virus [13], Semliki forest virus [14], human polyomavirus [15], human papillomavirus [16], echovirus [17], human immunodeficiency virus [18] and rotavirus [19]. These viruses typically utilize common molecules on the cell membrane to facilitate their invasion into cells. These molecules, including heparan sulfate proteoglycans (HSPGs), provide the first anchoring sites on the cell surface and help the virus make primary contact with host cells [20]. It has been shown that LF is able to prevent the internalization of some viruses by binding to HSPGs, which is present on most cells [21]. This property of LF confers protection to the host against viral infections. Based on these findings, we hypothesize that another underlying mechanism for the anti-SARS-CoV effect of LF involves its capability to bind to the extensively distributed HSPGs molecule on host cells. Our results indicate that HSPGs provide the preliminary docking sites on the cell surface and play an important role in the process of SARS pseudovirus cell entry. LF can block the infection of SARS pseudovirus by binding to HSPGs, suggesting that it may exert a protective role in host immune defense against SARS-CoV invasion.

## Materials and Methods

### Plasmids and cell lines

The plasmid pQCXIX, the SARS-CoV spike protein-encoding vector sh-2, and the gag/pol expression plasmid were kindly provided by Dr. Wenhui Li [22]. VSV-G plasmid encoding Vesicular stomatitis virus (VSV) G glycoprotein was kindly provided by Dr. Xiaozhong Peng (Peking Union Medical College). The HEK293E/ACE2-Myc, HEK293E/S1190-Fc and HEK293E/Fc cell lines, stably expressing ACE2-Myc, S1190-Fc and human IgG Fc fragment, respectively, were generated in our laboratory as described previously [22], [23]. The human colon carcinoma-derived Caco-2 cells and the African green monkey kidney-derived Vero E6 cells were provided by American Type Culture Collection (ATCC). Cells were grown in high-glucose Dulbecco's modified Eagle's medium (DMEM) (Hyclone) supplemented with 10% fetal bovine serum (FBS) at 37°C in humidified incubators with 5% CO_2_.

### Preparation of pseudotyped viruses

SARS pseudotyped viruses were produced as reported previously [22]. Briefly, HEK293T cells at 70% confluence in 10-cm dishes were co-transfected with 4 µg of pQCXIX, 2 µg of sh-2, and 4 µg of the gag/pol expression plasmid using Lipofectamine 2000 (Invitrogen). After a 48-hour transfection, viral supernatants were harvested and filtered through screens with 0.45-µm pore size. Viral particles without glycoprotein as negative control were prepared with pQCXIX and gag/pol plasmids by the same method as above. pQCXIX, gag/pol and VSV-G plasmids were used for VSV-G pseudotyped virus packaging in the same way.Viral stocks were aliquoted and frozen at −80°C for long-term storage. Short-term storage at 4°C did not dramatically affect viral titers. Viral titers were determined as previously described [24].

### Pseudovirus infection assay

A total of 2×10^5^ HEK293E/ACE2-Myc cells were seeded into 12-well plates. After 24 hours, the cells were washed with phosphate-buffered saline (PBS) two times and the culture was replaced with fresh DMEM without FBS. Bovine LF (Wako) or heparin (Sigma-Aldrich) at the indicated concentration was added to the cells at 37°C for 1 h or 10 min, respectively. Subsequently, 1×10^5^ transducing units (TUs) of SARS pseudoviruses were added to the cells and incubated at 37°C for 4 h. The concentration of LF or heparin was maintained throughout the process of viral infection. Simultaneously, 15 µM bovine serum albumin (BSA) (Sigma-Aldrich) or PBS was used as control by the same method as described above, respectively. Unbound pseudovirions were removed by three washes with PBS. Then, the cells were cultured with fresh DMEM with 10% FBS at 37°C for 48 h. The SARS pseudovirus-infected GFP-expressing HEK293E/ACE2-Myc cells were observed by fluorescence microscopy (Nikon Eclipse TE 2000-U) and counted by flow cytometry (Beckman Coulter Epics Elite EST). The tests of SARS pseudovirus on Vero E6 and Caco-2 cells were performed by the same method as described above. The infection of HEK293E/ACE2-Myc cells by VSV-G pseudotyped virus in the presence of LF or heparin was carried out in the same way. Viral particles without glycoprotein were incubated with the cells to test whether they had the capability of infection or not.

### Western blotting

HEK293E/ACE2-Myc cells (2×10^5^) were seeded into 12-well plates. The next day, after incubation at 37°C for 1 h with LF at concentrations of 0.34 µM, 1.3 µM, 4 µM and 12 µM, HEK293E/ACE2-Myc cells were treated with 1×10^5^ SARS pseudovirus particles for 4 h at 37°C. 15 µM BSA was used as control. Then, the pseudovirus-containing supernatant was removed, and the cells were washed three times with PBS. After growth in fresh DMEM with 10% FBS at 37°C for 48 h, the cells were harvested to analyze GFP expression level by western blotting. Band density was calculated from western blots using Quantity One software.

### S1190-Fc binding assay

S1190-Fc and human IgG Fc was prepared as described by Hongliang Wang et al. in our laboratory [22]. Briefly, S1190-Fc and Fc were expressed by HEK293E/S1190-Fc and HEK293E/Fc cells, respectively. S1190-Fc is a recombinant protein composed of the soluble extracellular region (amino acids 1–1190) of SARS spike protein with human IgG Fc fused to its C terminus. The proteins were purified using a protein A column (GE Healthcare), and the protein concentration was measured with a BCA assay kit from Bio-Rad. HEK293E/ACE2-Myc cells at 80% confluence were harvested from 10-cm dishes. HEK293E/ACE2-Myc cells (2×10^5^) were exposed to 5 nM S1190-Fc at 4°C for 1 h after incubation with LF or heparin at 37°C for 1 h or 10 min, respectively. The concentration of LF or heparin was maintained throughout the process of incubation. Fc protein was used as a control. Unbound S1190-Fc was removed by three washes with PBS. Cell surface bound S1190-Fc was detected with a FITC-labeled mouse anti-human IgG Fc secondary antibody (Santa Cruz). After three washes with PBS, the mean fluorescence intensity (MFI) of each group was measured by flow cytometry. The results were analyzed by FlowJo software.

### ELISAs

96-well immunosorbent plates were coated with 100 µl of 20 µg/ml S1190-Fc or Fc in sodium carbonate buffer (0.1 M NaHCO_3_, pH 8.6) at 4°C overnight, followed by blocking for 2 h at 37°C with 300 µl of 3% bovine serum albumin (BSA) in PBS. Wells coated with Fc and PBS were used as negative controls and blank controls, respectively. HEK293E/ACE2-Myc cells were lysed to harvest the ACE2-Myc containing supernatant. To determine whether LF interferes with the interaction between S1190-Fc and ACE2-Myc, both S1190-Fc and ACE2-Myc were treated with LF. 20 µM LF was added to the S1190-Fc coated wells and to the ACE2-Myc containing supernatant, respectively, at the same time and incubated for 1 h at 37°C. LF untreated ACE2-Myc and S1190-Fc were used as control. Then, the LF pretreated or untreated ACE2-Myc was added to S1190-Fc coated wells with or without LF at 37°C for 1 h to allow them to interact with each other. The wells were washed six times with PBST (phosphate-buffered saline with 0.1% Tween 20) and then incubated with 100 µl of mouse anti-Myc antibody (Santa Cruz) diluted 1∶500 in PBS for 1 h. After the plates were washed, horseradish peroxidase-conjugated goat anti-mouse antibody (Santa Cruz) was added into each well, incubated at 37°C for 1 h and washed six times with PBST. The plates were developed with 100 µl of tetramethylbenzidine substrate. The reaction was terminated with 50 µl of 2.0 M H_2_SO_4_, and the absorbance was read at 450 nm.

### Confocal microscopy

Oregon Green 488-labeled LF was prepared according to the manual provided by Invitrogen and used at a concentration of 0.5 µM in PBS (pH 7.4). HEK293E/ACE2-Myc cells were grown on coverslips in 24-well plates. After incubation with Oregon Green-labeled LF at 4°C for 1 h, unbound LF in the culture supernatant was removed by three washing steps. Then, the cells were fixed with 4% paraformaldehyde for 15 min. Subsequently, cell membrane and nuclei were stained with 1,1′-Dioctadecyl-3,3,3′,3′-tetramethylindocarbocyanine perchlorate (DiI) (Sigma-Aldrich) and Hoechst33342 (Sigma-Aldrich), respectively. The subcellular localization of LF was observed using confocal laser-scanning microscopy (Leica TCS SP2), and the images were analyzed using Leica confocal software.

### Enzymatic degradation of cell-surface heparan sulfate and chondroitin sulfate

Heparan sulfate was removed from the cell surface of HEK293E/ACE2-Myc cells by treatment with heparinase I (Sigma-Aldrich). Cells were grown to 80% confluence in 12-well plates, washed three times with DMEM without FBS, and incubated with 10 units of heparinase I per ml for 1 h at 37°C in heparinase I buffer (20 mM Tris–HCl, 50 mM NaCl, 4 mM CaCl_2_, 0.01% BSA, pH 7.5). After three washes with PBS, the cells were resuspended in fresh DMEM without FBS and subjected to the subsequent SARS pseudovirus, VSV-G pseudovirus or S1190-Fc treatment as described above. The same method was also applied in the treatment of Vero E6 or Caco-2 cells. Chondroitin sulfate on the cell surface were digested by Chondroitinase ABC (Sigma-Aldrich) in the same way.

### Statistical analysis

All data are presented as the mean±SD from at least three independent experiments. Statistical analyses were performed using Student's *t*-test. Differences with P<0.05 were considered significant.

## Results

### Lactoferrin inhibits entry of SARS pseudovirus into HEK293E/ACE2-Myc cells

In addition to replication-competent viruses, pseudoviruses have become an ideal tool to investigate cell entry of SARS-CoV without safety concerns [22], [25], [26]. SARS pseudovirus possesses the morphological characteristics of replication-competent SARS-CoV, with SARS-CoV spike protein on the envelope membrane, and can mimic SARS-CoV in the process of cell entry. We use three plasmids to produce the SARS pseudovirus particles: gag/pol, pQCXIX and sh-2. The gag/pol plasmid carries the lentiviral gene gag-pol for the expression of the capsid proteins and the enzymes for replication. pQCXIX encodes the lentiviral packaging signal and the gene for GFP. sh-2 is responsible for SARS spike protein expression. Coexpression of the three plasmids in HEK293T cells results in the incorporation of SARS-CoV spike protein into the budding lentiviral particle envelope along with the reporter GFP, enabling the analysis of spike-mediated entry into host cells.

It has been reported that LF is able to inhibit a broad range of viruses at the early attachment stage [27]. To establish the antiviral effects of LF on SARS-CoV, we utilized SARS pseudovirus and HEK293E/ACE2-Myc cells to perform a series of a transduction assays in the presence or absence of bovine LF. To facilitate the detection of SARS pseudovirus internalization, our laboratory has generated the cell line HEK293E/ACE2-Myc, which stably expresses surface-localized ACE2 with a Myc tag fused to its C terminus. As demonstrated in previous studies, the HEK293E/ACE2-Myc cell line is a perfect tool to study the interaction of SARS-CoV and host cells, particularly at the stage of SARS-CoV cell entry [22], [28], [29].

**Figure 1:**
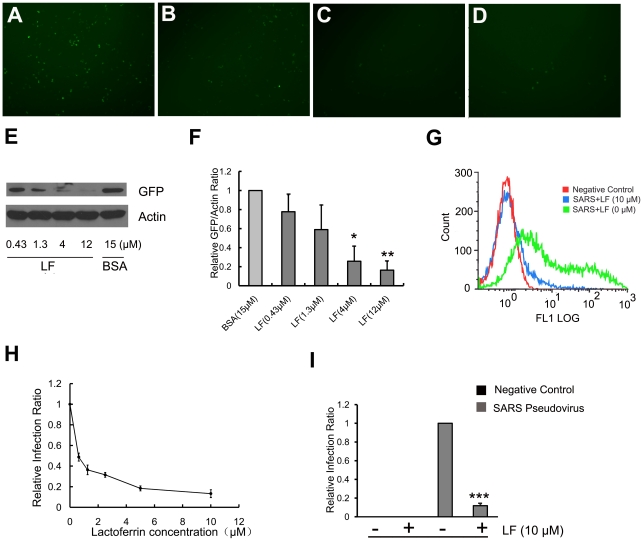
Lactoferrin inhibits SARS pseudovirus infection of HEK293E/ACE2-Myc cells. (**A–D**) Fluorescence microscopy illustrates that the number of SARS pseudovirus-infected GFP-expressing HEK293E/ACE2-Myc cells decreases in the presence of LF. HEK293E/ACE2-Myc cells were treated with LF for 1 h at 37°C at the concentration of 1 µM (B), 3 µM (C) or 10 µM (D). BSA (10 µM) was used as control (A). The LF-pretreated cells were treated with SARS pseudovirus as described in Methods. (**E and F**) Western blotting reveals that LF markedly reduces GFP expression in HEK293E/ACE2-Myc cells incubated with SARS pseudovirus. Statistical analysis of the relative band density ratio of GFP to actin was performed using a *t*-test. Error bars represent the SD of three independent experiments. **P<0.01 and *P<0.05. (**G and H**) Flow cytometry demonstrates that LF is able to inhibit the infection of HEK293E/ACE2-Myc cells by SARS pseudovirus. The concentration of LF was 0.625 µM, 1.25 µM, 2.5 µM, 5 µM or 10 µM. BSA (10 µM) served as control. The percentage of GFP expressing cells in the total population was calculated by flow cytometry. The relative viral infection ratio was measured by comparing the percentage of GFP expressing cells in each group with that of the BSA control. Error bars represent the SD of three independent experiments. (**I**) No GFP expression can be detected in the cells treated with viral particles without spike protein. The percentage of GFP expressing cells in the total population was calculated by flow cytometry as described above. Error bars represent the SD of three independent experiments.

We incubated HEK293E/ACE2-Myc cells with different concentrations of LF at 37°C for 1 h. Then SARS pseudoviruses were added to the LF-treated cells to test the effect of LF on SARS pseudovirus infection. As shown in Figure 1A–1D, the number of GFP expressing cells decreased sharply with increasing concentration of LF. Because cell entry of SARS pseudoviruses leads to GFP expression, this result suggests that the infection of HEK293E/ACE2-Myc cells by SARS pseudovirus can be dramatically inhibited in the presence of LF. We further utilized western blotting to examine GFP expression in the LF-treated cells after incubation with SARS pseudovirus. We found that the amount of GFP protein was reduced in the presence of LF (Fig. 1E and 1F). The degree of inhibition was correlated with the concentration of LF. At the same time, the GFP expressing cells were tested by flow cytometry after incubation with LF and SARS pseudovirus. Figures 1G and 1H demonstrate that LF inhibits SARS pseudovirus infection in a dose-dependent manner. The 50% inhibitory concentration (IC50) is approximately 0.7 µM. However, there was no GFP expression in the cells incubated with viral particles without spike protein (Fig.1I), indicating that SARS pseudovirus cell entry is mediated by spike protein. The data described above suggest that LF can prevent SARS pseudovirus from infecting host cells and that the inhibitory effect is dose dependent.

### Lactoferrin blocks spike protein binding to HEK293E/ACE2-Myc cells by an ACE2-independent pathway

**Figure 2:**
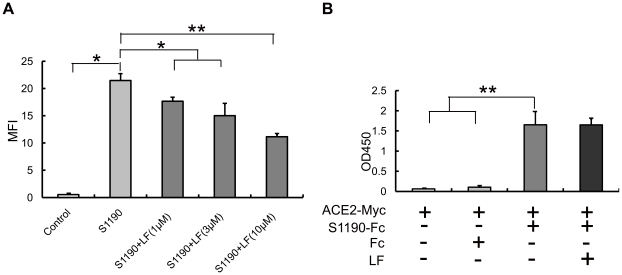
Lactoferrin blocks the interaction between spike protein and HEK293E/ACE2-Myc cells in an ACE2-independent fashion. (**A**) LF inhibits the binding of S1190-Fc to HEK293E/ACE2-Myc cells. Before incubation of S1190-Fc with HEK293E/ACE2-Myc cells at 4°C for 1 h, the cells were treated with LF at 37°C for 1 h at concentrations of 1 µM, 3 µM and 10 µM. Fc protein was used as a control. S1190-Fc binding to the cells was detected by flow cytometry as described in Methods. Error bars represent the SD of three independent experiments. ***P<0.001, **P<0.01 and *P<0.05. (**B**) LF does not disrupt the binding of S1190-Fc to ACE2-Myc. Error bars represent the SD of three independent experiments. ** P<0.01.

Previous studies have revealed that the infection of host cells by SARS-CoV is driven by spike protein, which is the only envelop protein responsible for attachment and fusion of the viral and the cellular membranes [30]. Thus, the preventive effect of LF on SARS pseudovirus infection may occur through targeting the attachment or fusion step. To determine whether the inhibitory effect was due to LF blocking the interaction between spike protein and host cells, we incubated S1190-Fc with the LF pre-treated HEK293E/ACE2-Myc cells at 4°C for 1 h. S1190-Fc is a soluble, truncated form of SARS CoV spike protein that retains the extracellular region (amino acids 1–1190) with human IgG Fc fused to its C terminus. After the treatment described above, the MFI of each group was analyzed by flow cytometry. As shown in Figure 2A, LF can effectively block S1190-Fc binding to HEK293E/ACE2-Myc cells at 4°C, suggesting that LF exerts its inhibitory effect on SARS pseudovirus internalization at the initial attachment stage.

The cell entry of SARS CoV is a rather complex process triggered by the interaction of spike protein with some receptors and/or cofactors on the cell surface. It has been established that ACE2 serves as a functional receptor for SARS-CoV infection [30]. The lectin DC-SIGN (dendritic cell-specific ICAM-grabbing non-integrin), or other related molecules, may also play an important role in SARS-CoV invasion [31]. To date, the underlying mechanism of viral entry is not fully understood, and further investigations are needed to elucidate this subtle process. To test whether LF prevents spike protein binding to the main receptor ACE2, we added LF to S1190-Fc and ACE2-Myc containing supernatants. After incubation at 37°C for 1 h, we mixed these solutions together in immunosorbent plates to examine the interaction between S1190-Fc and ACE2-Myc by ELISA. The results suggest that LF does not disrupt the binding of S1190-Fc to ACE2-Myc (Fig. 2B). Thus, LF may employ other mechanisms to inhibit the attachment of SARS pseudovirus to host cells.

### Subcellular localization of lactoferrin

**Figure 3:**
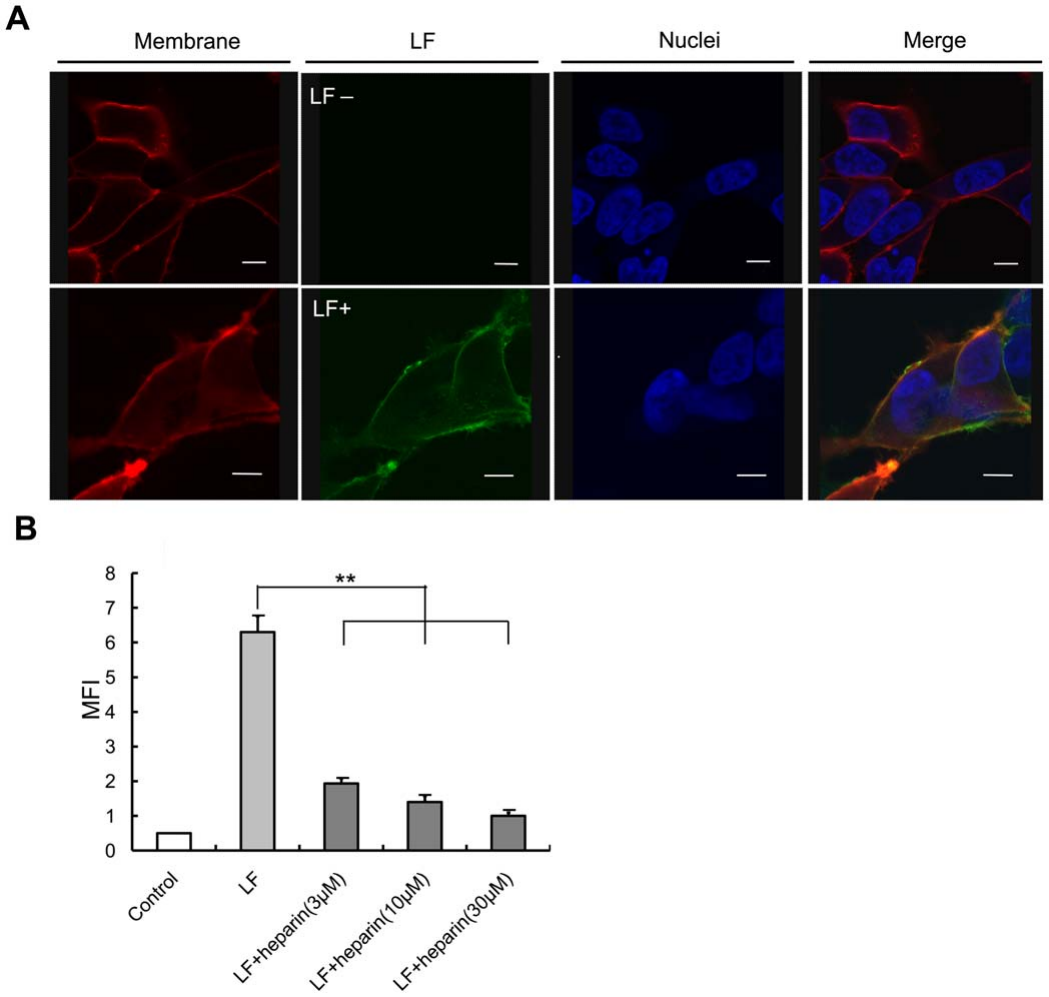
Lactoferrin localizes to the cell membrane by targeting HSPGs. (**A**) LF is present on the cell surface. Oregon Green-labeled LF localization was observed under confocal microscopy. LF untreated cells were used as control. Cell membrane and nuclei were stained with DiI and Hoechst33342, respectively. Scale bar, 8 µm. (**B**) Heparin inhibits LF binding to HEK293E/ACE2-Myc cells. HEK293E/ACE2-Myc cells were incubated with 0.5 µM Oregon Green-labeled LF at 4°C for 1 h after pretreatment with heparin at the concentration of 3 µM, 10 µM or 30 µM. The MFI was measured for each group by flow cytometry as described above. Error bars represent the SD of three independent experiments. **P<0.01.

Spike protein is the protein on the SARS-CoV envelope responsible for entry into host cells. The binding of spike protein to the ACE2 receptor can initiate fusion between the viral and cellular membranes [6]. Based on these facts, we deduced that if LF did not block the binding of spike protein to ACE2, it could interact with another molecule on the cell surface, thereby playing an inhibitory role against SARS pseudovirus cell entry. To determine the subcellular localization of LF, we labeled LF with Oregon Green fluorescent dye and incubated the labeled LF with HEK293E/ACE2-Myc cells. At the same time, cell membrane was stained with the red membrane dye DiI. According to the confocal microscopy results, LF was present on the cell membrane (Fig. 3A).

Previous studies have established that LF mediates inhibition of some viral infections by interfering with virus-cell interactions after binding to the widespread family of cell-surface molecules, the HSPGs [32]–[34]. HSPGs are complex macromolecules consisting of unbranched heparan sulfate (HS) polysaccharide chains, composed of repeating disaccharide subunits of hexuronic acid and hexosamine, covalently linked to the core protein through O-glycosidic linkages [20]. The binding of LF to HSPGs prevents the first contact between virus and host cells and thus prevents subsequent infection. To ascertain whether LF interacts with the cell-surface HSPG molecules, we used heparin to interfere with the binding of LF to target cells. Due to their similar structural compositions, exogenous heparin has been widely utilized as a soluble HS analog to competitively inhibit the interaction between HSPGs and their ligands [35]. Prior to incubation with Oregon Green-labeled LF, HEK293E/ACE2-Myc cells were treated with heparin at different concentrations. The MFI was analyzed to detect the amount of LF binding for each group. As shown in Figure 3B, exogenous heparin efficiently prevented LF binding to HEK293E/ACE2-Myc cells, indicating that LF localizes to the cell surface mainly through interactions with HSPGs.

### Lactoferrin inhibits spike protein binding to HEK293E/ACE2-Myc cells and SARS pseudovirus infection by binding to cell-surface HSPGs

**Figure 4:**
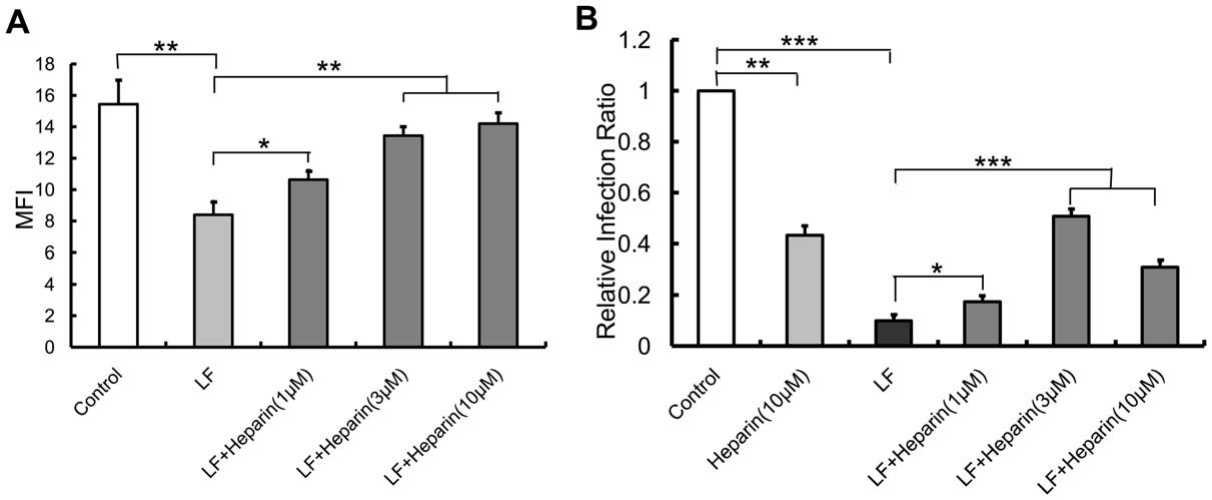
Lactoferrin exerts its inhibitory effect on spike protein and SARS pseudovirus by binding to cell-surface HSPGs. (**A**) Heparin neutralizes the LF-mediated inhibition of S1190-Fc binding to HEK293E/ACE2-Myc cells. Before treatment with 10 µM LF for 1 h at 37°C, HEK293E/ACE2-Myc cells were incubated with heparin for 10 min at the concentration of 1 µM, 3 µM or 10 µM. S1190-Fc was added to each group to detect S1190-Fc binding to the cells as described above. Error bars represent the SD of three independent experiments. ***P<0.001, **P<0.01 and *P<0.05. (**B**) Inhibition of SARS pseudovirus infection of HEK293E/ACE2-Myc cells by LF can be partially neutralized by heparin. HEK293E/ACE2-Myc cells were treated with heparin for 10 min at the concentration of 1 µM, 3 µM or 10 µM. Then, 10 µM LF was added to each group and incubated at 37°C for 1 h. The GFP-expressing HEK293E/ACE2-Myc cells in the total population were analyzed as described above. The relative viral infection ratio was measured by comparing the percentage of GFP expressing cells of each group with that of the BSA control. Error bars represent the SD of three independent experiments. *** P<0.001, ** P<0.01 and *P<0.05.

To further investigate whether LF inhibits S1190-Fc binding to HEK293E/ACE2-Myc cells due to its binding to cell-surface HSPGs, we added S1190-Fc after incubating heparin pre-treated HEK293E/ACE2-Myc cells with LF. Figure 4A shows that heparin neutralized the inhibitory effect of LF on the binding of S1190-Fc to host cells in a dose-dependent manner, suggesting that the anchoring sites for LF on the cell surface are provided by the HS moieties of HSPGs. The HS analog heparin competed for LF binding to HSPGs, preventing cell binding.

In the next experiment, we examined whether heparin could neutralize the LF-mediated inhibition of SARS pseudovirus infection of HEK293E/ACE2-Myc cells in the same manner. Prior to SARS pseudovirus infection, we added heparin to the cells and incubated the heparin-treated cells with LF for 1 h at 37°C. As shown in Figure 4B, adding heparin increased the infectivity of SARS pseudovirus in the presence of LF compared with controls. This evidence further supports the conclusion that LF blocks adsorption and internalization of SARS pseudovirus through binding to HSPGs on the cell surface. Interestingly, another phenomenon also captured our attention. Adding heparin alone also prevented the cell entry of SARS pseudovirus into host cells (Fig. 4B). This result is consistent with the previous finding that heparin can reduce the infection of Vero E6 cells by replication-competent SARS-CoV [36]. This evidence implies that HSPGs play an important role in SARS-CoV cell entry. Furthermore, we found that, instead of increasing cell entry, incubating HEK293E/ACE2-Myc cells with 10 µM heparin and 10 µM LF curtailed the infectivity of SARS pseudovirus, compared with the cells treated with 3 µM heparin and 10 µM LF. One reasonable explanation for this result is that excessive heparin competes with the HS chain on the cell surface and prevents adsorption of SARS pseudovirus to host cells. These data indicate that HSPGs are important molecules involved in SARS pseudovirus cell entry.

### HSPGs provide docking sites for spike protein on the cell surface and play an important role in SARS pseudovirus infection

**Figure 5:**
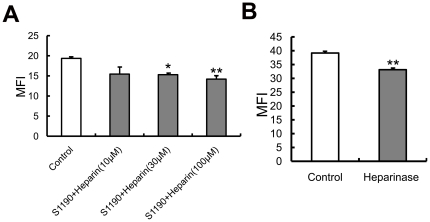
Addition of exogenous heparin and enzymatic removal of HS chains by heparinase reduce spike protein binding to HEK293E/ACE2-Myc cells. (**A**) Heparin blocks the binding of S1190-Fc to HEK293E/ACE2-Myc cells. HEK293E/ACE2-Myc cells were incubated with heparin for 10 min at the concentration of 10 µM, 30 µM or 100 µM. S1190-Fc was added to each group and incubated at 4°C for 1 h. The MFI of each group was measured as described above. Error bars represent the SD of three independent experiments. *** P<0.001, **P<0.01 and *P<0.05. (**B**) Enzymatic degradation of cell-surface heparan sulfate (HS) chains reduces S1190-Fc binding. After treatment with 10 U of heparinase I, the cells were incubated with S1190-Fc at 4°C for 1 h. The MFI test was performed using the same method as above.

To further prove the involvement of HSPGs in the SARS-CoV entry process, we first utilized S1190-Fc to test whether HSPGs participated in the viral adsorption step. Prior to incubation with S1190-Fc at 4°C for 1 h, HEK293E/ACE2-Myc cells were treated with heparin for 10 min. The mean fluorescence intensity analysis demonstrates that the binding of S1190-Fc to the cells was efficiently blocked by the addition of heparin (Fig. 5A). Then, we used heparinase I to degrade the HS chain on the cell surface. We found that enzymatic removal of HS polysaccharides resulted in a reduced ability of S1190-Fc to bind to host cells at 4°C (Fig. 5B), suggesting that in addition to ACE2, HSPGs act as another docking site for SARS-CoV on the cell surface. The presence of ACE2 and heparinase-resistant oligosaccharides may give rise to the incomplete inhibition [37].

**Figure 6:**
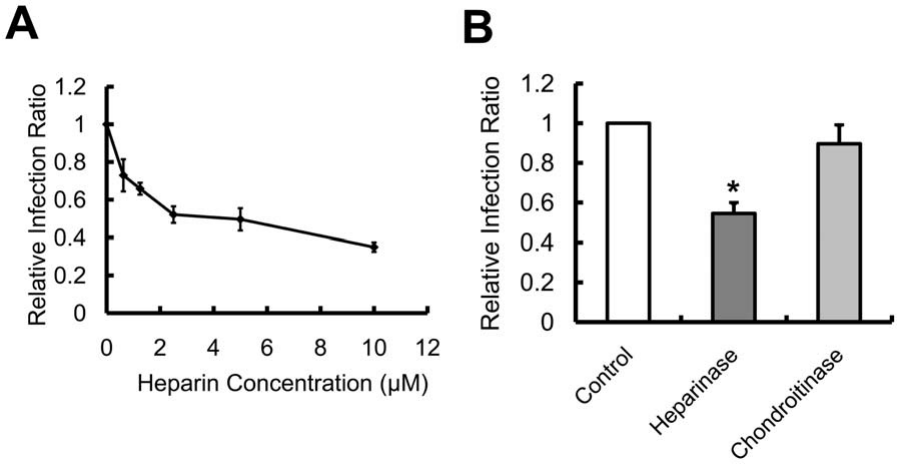
Incubation with heparin and degradation of HS polysaccharides on the cell surface by heparinase inhibits SARS pseudovirus infection of HEK293E/ACE2-Myc cells. (**A**) Heparin inhibits SARS pseudovirus entry into HEK293E/ACE2-Myc cells. Before incubation with SARS pseudovirus at 37°C for 4 h, HEK293E/ACE2-Myc cells were treated with heparin for 10 min at the concentration of 0.625 µM, 1.25 µM, 2.5 µM, 5 µM or 10 µM. GFP-expressing HEK293E/ACE2-Myc cells in the total population were analyzed by flow cytometry. The relative viral infection ratio was measured by comparing the percentage of GFP expressing cells of each group to that of the BSA control. Error bars represent the SD of three independent experiments. (**B**) Lysis of cell-surface HS by heparinase blocks the infection of HEK293E/ACE2-Myc cells by SARS pseudovirus. After incubation with 10 U of heparinase I or chondroitinase ABC for 1 h at 37°C, the cells were treated with SARS pseudovirus as described above. The relative viral infection ratio was calculated by the same method. *P<0.05.

To provide further supporting evidence of the requirement for cell-surface HSPGs during SARS-CoV infection, we added heparin to HEK293E/ACE2-Myc cells before incubating the cells with SARS pseudovirus. The treatment of host cells with a cellular HS analog dramatically decreased the infectivity of SARS pseudovirus (Fig. 6A), indicating that SARS pseudovirus cell entry is blocked by competitive inhibition on the part of heparin and that the binding sites provided by HSPGs are important for effective infection. The enzymatic degradation of HS chains from the cell surface also led to resistance of HS-deficient cells to SARS pseudovirus infection (Fig. 6B), demonstrating that the HS chains of HSPGs are involved in SARS pseudovirus cell entry. Because HSPGs are widely distributed on the cell membrane, it is very likely that they serve as the initial anchoring site, facilitating the preliminary contact between the virus and host cells and the subsequent concentration of viruses on the cell surface. According to previous studies, viral particles are then transferred from the low affinity HSPGs to specific high affinity entry receptors, which results in internalization into host cells [37]. HSPGs themselves are not sufficient for SARS-CoV entry. HSPGs are widely expressed on most mammalian cells. However, SARS-CoV can be found only in limited host cells, such as lung alveolar epithelial cells, enterocytes of the small intestine and the brush border of the proximal tubular cells of the kidney [3]. Therefore, HSPGs may play an important role in promoting SARS-CoV infection by facilitating access to a specific entry receptor. However, when chondroitin sulfate was removed from cell surface by enzymatic digestion, SARS pseudovirus could still effectively infect the host cells, suggesting that compared with HSPG, chondroitin sulfate may have much less impact on SARS pseudovirus cell entry.

**Figure 7:**
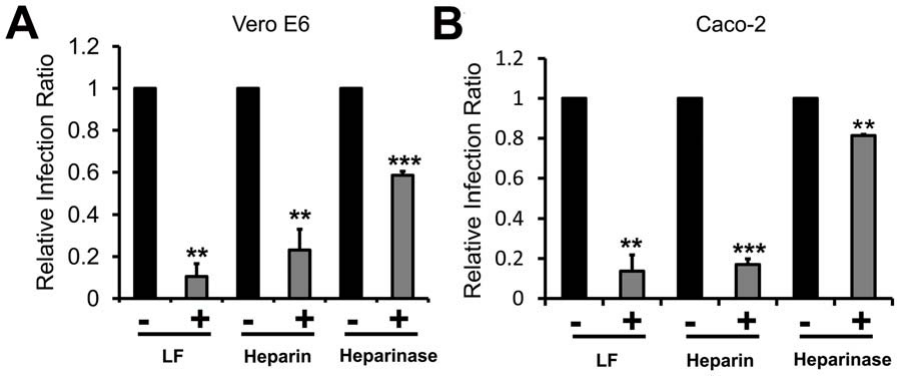
LF, heparin or enzymatic removal of cell surface HSPGs can prevent SARS pseudovirus entry into Vero E6 or Caco-2 cells. (**A**) Interference of the interaction between Vero E6 and SARS pseudovirus by LF, heparin and heparinase leads to reduction of viral infection. Vero E6 cells were treated by the same methods above with 10 µM LF, 10 µM heparin or 10 U of heparinase I, respectively. Then, the cells were incubated with SARS pseudovirus as described in Methods. GFP-expressing cells in the total population were analyzed by flow cytometry. The relative viral infection ratio was measured by comparing the percentage of GFP expressing cells of each group to that of the control. Error bars represent the SD of three independent experiments. *** P<0.001 and **P<0.01. (**B**) Incubation with LF or heparin, or degradation of HSPGs by heparinase inhibits SARS pseudovirus infection of Caco-2 cells. The Caco-2 cells were treated by same methods as described above. *** P<0.001, **P<0.01 and *P<0.05.

We further investigated the role of HSPGs in SARS pseudovirus cell entry into Caco-2 and Vero E6 cells. As shown in Figure 7, SARS pseudovirus infection was efficiently blocked by LF, heparin or cell surface HSPGs degradation, respectively. Again, these results provide further supporting evidence of the involvement of HSPGs in the SARS-CoV cell entry.

**Figure 8:**
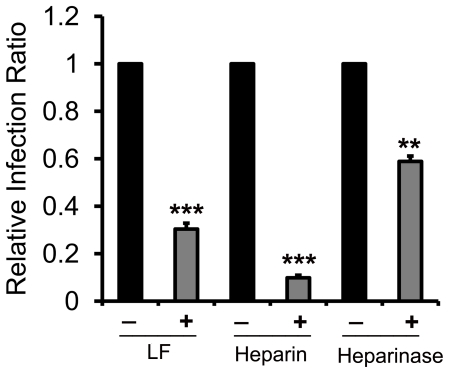
HSPGs are also involved in VSV-G virus cell entry. HEK293E/ACE2-Myc cells were treated with 10 µM LF, 10 µM heparin or 10 U of heparinase I, respectively, in the same way. And the same methods were used for the subsequent assay as described above. GFP-expressing cells in the total population were analyzed by flow cytometry. The relative viral infection ratio was measured by comparing the percentage of GFP expressing cells of each group to that of the control. Error bars represent the SD of three independent experiments. *** P<0.001 and **P<0.01.

VSV-G pseudotyped virus was usually used as control in other virus studies. Thus, the role of HSPGs in VSV-G pseudotyped virus cell entry was investigated in our experiments. Surprisingly, we found that soluble heparin and enzymatic removal of cell surface HSPGs effectively inhibited VSV-G virus particle infection (Fig. 8), which is consistent with previous reports using replication-competent VSV and VSV-G pseudotyped virus on other cell lines, including human Hela, avian CER, human bronchial 16HBE-S1 and tracheal CFT1-C2 epithelial cells, etc. [38]–[41]. Furthermore, previous reports have shown that LF could exert an inhibitory effect on VSV infection [42], which is also confirmed by our experiment result (Fig. 8). All these data suggest that HSPGs also play an important role in the process of VSV cell entry. The binding of LF to cell surface HSPGs may account for the reduction of VSV infection. To date, many investigations have been performed to elucidate this rather complicated process of viral cell entry. To sum up the findings of the underlying mechanism of viral infection, we speculated that it might be an universal phenomenon that enveloped viruses tend to utilize cell-surface HSPGs as primary anchoring sites to initiate the subsequent cell entry.

## Discussion

Recent studies have revealed that viral entry is a highly complex process that usually involves various molecules on the cell surface [43]. The primary step is often initiated by low-affinity binding to attachment sites, which promotes the concentration of virions on the cell surface. The subsequent binding to a high-affinity receptor triggers cell entry [44], [45]. The ubiquitous HSPGs, which are widely distributed on mammalian cells, have been identified as the initial docking site for a number of viruses, such as herpes virus [46], hepatitis C virus [47], dengue virus [48], human immunodeficiency virus type 1 [20], foot and mouth disease virus [20], human papillomavirus [49] and hepatitis B virus [50]. It has been revealed that HSPGs acts as primary binding sites, promoting viral docking and facilitating subsequent interaction with the specific receptors. Furthermore, HSPGs can function as storage sites on non-permissive cells and mediate “in trans” infection by presenting the viruses to their target cells [51].

**Figure 9:**
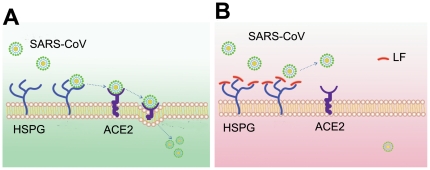
A model of SARS-CoV cell entry and the protective role of Lactoferrin in SARS-CoV infection. (**A**) HSPGs play an important role in the process of SARS-CoV cell entry. The anchoring sites provided by HSPGs permit initial contact between SARS-CoV and host cells and the concentration of virus particles on cell surface. SARS-CoV rolls onto the cell membrane by binding to HSPGs and scans for specific entry receptors, which leads to subsequent cell entry. (**B**) LF blocks the infection of SARS-CoV by binding to HSPGs. LF expression may be up-regulated when SARS-CoV infects the human body. LF locates to cell-surface HSPGs and prevents the preliminary interaction between the virus and host cells and the subsequent internalization process.

Previous studies have indicated that in addition to ACE2, other coreceptors or cellular molecules are required for SARS-CoV infection [7]. Our results suggest that the efficient entry of SARS-CoV into host cells requires the involvement of HSPGs in concert with ACE2. Increased infectivity of SARS pseudovirus was associated with binding to HS. Elimination of cell-surface HS by heparinase or the addition of exogenous heparin reduced the ability of the virus to bind to the cell surface and increased cellular resistance to infection. Furthermore, it has been demonstrated that murine coronavirus utilizes HSPGs as a receptor for cell entry [52], suggesting that HSPGs may also play an important role in the process of SARS-CoV infection due to the similarity of the spike protein structures of these two viruses. Thus, it is very likely that HSPGs serve as SARS-CoV attachment sites and facilitate the concentration of the virus on the cell surface as well as access to specific entry receptors. At the preliminary stage, the docking of the virus to host cells is mediated by the interaction between the spike protein and HS chains of HSPGs. This association facilitates the further binding of SARS-CoV to its cell-surface receptor, ACE2. The current widely accepted model for the role of HSPGs suggests that the transport of extracellular virus particles from the low affinity anchoring sites to the high affinity specific entry receptors is a ubiquitous phenomenon in viral invasion, which is termed ‘viral surfing’ [48]. Through the weak and reversible interaction between the virus and the docking sites, the virus binds to host cells and scans the cell surface for the specific receptors. SARS-CoV may also adopt a similar strategy for cell entry. Figure 9 illustrates the hypothetical process of SARS-CoV infection. However, SARS-CoV attachment to HSPGs alone does not enable viral entry. HSPGs are widely distributed on the surface of mammalian cells. However, the target cells of SARS-CoV are limited to lung alveolar epithelial cells, enterocytes of the small intestine and the brush border of the proximal tubular cells of the kidney, among others [3]. Therefore, HSPGs play an important and complex role in promoting SARS-CoV infection. The entry process also requires the interaction of viral spike protein with one or more co-receptor molecules, such as ACE2, on the cell surface.

It has been reported that SARS-CoV can utilize DC-SIGN to enhance infectivity [31]. Studies have confirmed that LF blocks the interaction between the DC-SIGN receptor on dendritic cells and HIV glycoprotein 120, resulting in inhibition of subsequent virus transmission [53]. These findings suggest that LF may prevent SARS-CoV spread in the human body through the same mechanism.

It has been revealed that LF is highly up-regulated (by 150 fold) in the peripheral blood mononuclear cells of SARS patients [8]. LF is usually present in plasma and external secretions and exerts a wide variety of biological functions, such as inhibiting infection by microbes, including bacteria, viruses, protozoa and fungi. LF is a highly conserved protein, with approximately 70% sequence homology between the bovine and human forms [54]. Therefore, bovine LF is often utilized as a substitute for human LF because of their similar functions [55], [56]. Our results suggest that LF can inhibit the entry of SARS pseudovirus into HEK293E/ACE2-Myc cells in a dose-dependent manner. LF does not disrupt the interaction between the spike protein and the ACE2 receptor *in vitro*. However, LF inhibits spike protein binding to HEK293E/ACE2-Myc cells. The target molecules on the cell membrane that LF interacts with are HSPGs. Previous three-dimensional structure analyses have revealed that LF carries highly cationic charge on its molecular surface, particularly in the N-terminal domain [57]. The highly positive surface charge is a characteristic feature of this multifunctional protein. Many studies have demonstrated that LF binds to HSPGs on the cell membrane with a high affinity through its N-terminal glycosaminoglycan-binding domain [58], [59]. HSPGs are composed of a core protein that is covalently connected with linear, polysulfated heparan sulfate polysaccharides. Due to their abundant carboxyl and sulfate groups, HSPGs are a major source of the highly negatively charged macromolecules that surround almost all mammalian cells [51]. This strong net negative charge permits HSPGs to bind to the N-terminal glycosaminoglycan-binding domain of LF through electrostatic attraction [58]. Our experiments revealed that LF-mediated inhibition of SARS-CoV infection occurs through LF competitively localizing to the SARS-CoV anchoring sites provided by HSPGs. The binding of LF to HSPGs prevented preliminary contact between SARS-CoV and host cells and, thus, prevented subsequent cell entry. In summary, LF may exert its protective functions against SARS-CoV invasion in two ways at the same time. On the one hand, as reported in previous research, LF enhances NK cell activity and stimulates neutrophil aggregation and adhesion in immune defense [8]; on the other hand, LF curtails the entry of SARS-CoV into host cells during SARS-CoV infection. LF may have potential therapeutic applications as a drug candidate for the treatment of SARS disease.

Since the SARS outbreak in November 2002, there is still no effective preventive vaccine or antiviral therapeutic strategy available to combat this deadly virus [59]. Although great progress has been made in understanding the molecular mechanism of the SARS-CoV life cycle, further investigations should be performed to elucidate the subtle process of SARS-CoV cell entry, which will provide deeper insight into rational drug design for the treatment of SARS disease.
